# Molecular identification and chromosomal localization of new powdery mildew resistance gene *Pm11* in oat

**DOI:** 10.1007/s00122-019-03449-3

**Published:** 2019-09-30

**Authors:** Tomasz Ociepa, Sylwia Okoń, Aleksandra Nucia, Justyna Leśniowska-Nowak, Edyta Paczos-Grzęda, Maciej Bisaga

**Affiliations:** 1 Institute of Plant Genetics, Breeding and Biotechnology, University of Life Sciences in Lublin, Akademicka 15, 20-950 Lublin, Poland; 2 Department of Cell Biology, University of Silesia in Katowice, Jagiellońska 28, 40-032 Katowice, Poland

## Abstract

The appropriate selection of various traits in valuable plants is very important for modern plant breeding. Effective resistance to fungal diseases, such as powdery mildew, is an example of such a trait in oats. Marker-assisted selection is an important tool that reduces the time and cost of selection. The aims of the present study were the identification of dominant DArTseq markers associated with a new resistance gene, annotated as *Pm11* and derived from *Avena sterilis* genotype CN113536, and the subsequent conversion of these markers into a PCR-based assay. Among the obtained 30,620 silicoDArT markers, 202 markers were highly associated with resistance in the analysed population. Of these, 71 were selected for potential conversion: 42 specific to resistant and 29 to susceptible individuals. Finally, 40 silicoDArT markers were suitable for primer design. From this pool, five markers, 3 for resistant and 2 for susceptible plants, were selected for product amplification in the expected groups. The developed method, based on 2 selection markers, provides certain identification of resistant and susceptible homozygotes. Also, the use of these markers allowed the determination of heterozygotes in the analysed population. Selected silicoDArT markers were also used for chromosomal localization of new resistance genes. Five out of 71 segregating silicoDArT markers for the *Pm11* gene were found on the available consensus genetic map of *oat*. Five markers were placed on linkage groups corresponding to Mrg12 on the *Avena sativa* consensus map.

## Introduction

Powdery mildew caused by the biotrophic fungus *Blumeria graminis,* an ascomycete belonging to the Erysiphales (Takamatsu 2004), is one of the most common and destructive diseases of cereals, including common oat (Hau and de Vallavieille-Pope 2006; Dean et al. 2012). Powdery mildew reduces grain yield by 10–39% in years of low and high disease pressure, respectively (Lawes and Hayes 1965; Jones et al. 1987). It also leads to reductions in grain protein content and a specific weight (Roderick et al. 2000). The disease appears in cold and humid regions, where rain occurs early in the season and temperatures are relatively low (Bennett 1984; Roderick et al. 2000). This has been reported as a serious problem in the UK (Roderick et al. 2000), north-western and central Europe (Schwarzbach and Smith 1988; Okoń 2012) and North America (Leath 1991). Also, the disease has spread in recent years to areas where it has not occurred previously, for example China (Xue et al. 2017) and the north-western Himalaya region (Banyal et al. 2016).

Resistance to powdery mildew is not widespread in *Avena sativa* (Hsam et al. 1997, 1998; Sánchez-Martín et al. 2011; Okoń 2012). Control of powdery mildew can be achieved through fungicide application, crop rotation and use of resistant cultivars (Martinelli 2004). The first method is ecologically undesirable, and the use of fungicides may lead to rapid adaptation of pathogens and insensitivity to the chemicals applied. Therefore, introducing effective resistance genes into cultivars via crossing with resistant genotypes is the most effective and environmentally friendly method of controlling this disease (Stevens et al. 2004).

Many research studies have focused on the identification and introduction of new resistance genes from lower-ploidy species, including diploids (Thomas 1992; Morikawa 1995) and tetraploids (Aung et al. 1977; Okoń et al. 2018b), but hexaploid species are also a valuable source of desirable traits (Lawes and Hayes 1965; Hoppe and Kummer 1991; Roderick et al. 2000; Okoń et al. 2016b).

Marker-assisted selection (MAS) is a method of selecting desirable individuals in breeding programmes based on DNA molecular marker patterns associated with particular traits, and it combines knowledge about the genotype and phenotype of the analysed plants (Collard and Mackill 2008). One of the main advantages of this method of selection is that it can be carried out at an early stage of plant growth (plantlets). Therefore, it has the potential for efficient gene pyramiding, i.e., combining several important genes in one cultivar. A wide group of molecular markers, including RFLP (Pal et al. 2002), RAPD (Penner et al. 1993), AFLP (Barbosa et al. 2006), SSR (Li et al. 2000; Becher 2007), SNP (Chen et al. 2006) and silicoDArT (Okoń et al. 2018a) have been successfully applied in the selection of valuable oat individuals.

The low genetic diversity of oats enforces the use of marker systems that identify high levels of polymorphism in the largest area of the genome (Paczos-Grzęda et al. 2014). DArTseq, which is a modification of the classical DArT method, is an example of such a high-throughput genotyping method. It consists of replacing the microarray hybridization step with next-generation sequencing in the Illumina system (Kilian and Graner 2012). As a result, two sets of markers are obtained: dominant and more numerous silicoDArTs and co-dominant and more informative SNPs (Milczarski et al. 2016).

The aim of the current study was characterization and chromosomal localization of new oat powdery mildew-resistant gene from *A. sterilis*, designed *Pm11*, as well as identification of dominant silicoDArT markers linked with this gene and conversion of these markers into a PCR-based assay.

## Materials and methods

### Plant material

The subjects of the study were F_2_ and F_3_ populations derived from the cross between the susceptible cultivar ‘Sam’ and *A. sterilis* genotype CN113536. This genotype has been identified as a valuable source of resistance against oat powdery mildew (Okoń et al. 2016b) and characterized as highly effective against the oat powdery mildew pathotypes present in Poland in 2010–2017 (Okoń and Ociepa 2018). Totally, 146 individuals from the *F*_2_ population were phenotyped based on the host–pathogen test. After testing, all individuals were planted in the experimental plot. F_3_ generation seeds were collected from each *F*_2_ individual. At least 15 plants representing one *F*_2_ individual were tested using a host–pathogen methodology to identify heterozygotes and homozygotes. Ninety-two genotypes from the *F*_2_ population and parental forms were used for genotyping based on the DArTseq method. The outcomes allowed the selection of both homozygous-resistant and homozygous-susceptible individuals for molecular analysis.

### Host–pathogen tests

To determine the resistance of individuals in the *F*_2_ and *F*_3_ populations, 146 individuals of each population were tested using two isolates of powdery mildew with different degrees of virulence. The selected isolates were collected from different parts of Poland, the first from Białka in the Świętokrzyskie Voivodeship and the second from Laski in the Mazovian Voivodeship, both in 2014. Selected isolates were obtained according to the methodology described by Okoń and Kowalczyk (2012a). Host–pathogen tests were carried out on the first leaves of 10-day-old seedlings. After 10 days of incubation, the results were scored and classified. Reactions to the isolates were grouped into two classes: resistant, from 0 to 20% infection relative to ‘Sam’, and susceptible where the degree of infection exceeded 20%. The segregation ratio of *F*_2_ and *F*_3_ populations was analysed using chi-square tests of goodness of fit. The results of the host–pathogen tests allowed us to determine the genetic basis of resistance derived from *A. sterilis*.

### DNA extraction

Genomic DNA from all *F*_2_ individuals was extracted from fresh 10-day-old leaves using the DNeasy Plant Mini Kit (Qiagen). DNA integrity and quality were evaluated by electrophoresis on a 1.5% agarose gel. The DNA concentration was determined with NanoDrop 2000 spectrophotometry and normalized to 50 ng μl^−1^.

### High-throughput genotyping using the DArTseq method

A high-throughput genotyping method based on DArTseq technology was used to genotype 92 individuals from the *F*_2_ population. The silicoDArT markers were scored as binary data (0/1) using DArTsoft, and several quality parameters such as call rate, polymorphism information content (PIC) and reproducibility were calculated.

### Conversion of silicoDArT markers to PCR-based assay

The silicoDArT markers highly correlated with phenotypic observations were selected for further analysis. Marker sequences associated with resistance to oat powdery mildew were analysed using the CLC Main Workbench software version 7.9.1 to identify primer pairs for their amplification. The main criteria for primer design were as follows: primer size 14–22 bp, GC content 40–60% (optimum 50%), minimum melting temperature 48 °C and product size > 40 bp.

### Specific PCR

Reaction mixtures had a final volume of 10 μl consisting of 60 ng of total genomic DNA, 20–40 μM of each PCR primer (quantity was measured for each primer), 0.1 mM dNTPs, 1.5–2.5 mM MgCl_2_ (quantity was tested for each primer), 1 × reaction buffer and 0.5 U Taq Polymerase, Thermo Fisher. PCR was conducted in a T1Biometra thermocycler. The following reaction profile was applied: 95 °C—7 min, 35 cycles (95 °C—30 s, *X* °C—30 s, where *X* temperature is the annealing temperature, determined empirically based on the calculation of the average melting temperature of the primer pair minus 2°–5°, 72 °C—30 s), with final elongation at 72 °C—5 min. PCR products were separated in a 2.5% agarose gels containing EtBr in TBE buffer at 140 V for 1 h.

### Marker validation

Converted silicoDArT-based markers were tested in 92 individuals from the F_2_ segregating population ‘Sam’ × CN113536 subjected to DArTseq genotyping. Segregation evaluated based on silicoDArT markers and their converted counterparts was compared for congruency. Spearman rank correlation coefficients between converted markers and original silicoDArT profiles were calculated using the Statistica software 13.1 (StatSoft 2017).

Markers with the highest correlation with silicoDArT profiles were tested on 146 phenotyped individuals from the Sam × CN113536 population. Spearman rank correlation coefficients between markers and phenotypic observation were calculated using the Statistica software 13.1 (StatSoft 2017).

The significance of association between the evaluated molecular profiles and resistant and susceptible plants was assessed by the Pearson chi-square test (Bewick et al. 2004) using the Statistica software 13.1 (StatSoft 2017).

### Chromosomal localization

Markers with the expected segregation for resistant and susceptible plants were used for chromosomal assignment analysis. Seventy-one segregating silicoDArT sequences were used to perform local BLASTn of segregating silicoDArT marker sequence against Chaffin et al.’s (2016) oat consensus map uploaded by Bekele et al. (2018) and were performed using the CLC Genomic Workbench version 8.0.1 with the lowest *e* value = 1*e*−10 and greatest identity ≥ 95%. The genetic position of silicoDArT markers was assigned based on their counterparts placement on the consensus map.

## Results

A total of 146 individuals of the ‘Sam’ × CN113536 *F*_2_ population were tested in 2 independent host–pathogen tests with 2 different powdery mildew isolates. In both tests, segregation for resistant and susceptible plants was obtained. The numbers of resistant and susceptible individuals were very similar: 104 and 103 resistant and 42 and 43 susceptible individuals were identified in the tests based on the Białka and Laski isolates, respectively. The segregation ratio of the *F*_2_ populations was analysed using chi-square tests for goodness of fit. In both cases, the observed ratio did not deviate from that expected under the model 3 resistant/1 susceptible plant at a *p* value of 5%; this value was 0.3 and 0.2 for Białka and Laski, respectively (Table 1). To confirm the monogenic inheritance of resistance, host–pathogen tests were carried out on individuals of the F_3_ population. The obtained segregation approximated a 1:2:1 ratio corroborating single-gene segregation (Table 1).

**Table 1:** Seedling responses and segregation ratios of *F*_2_ and *F*_3_ families derived from the ‘Sam’ × CN113536 cross inoculated with different *Blumeria graminis* DC. f. sp. *avenae* Em. Marschal isolates

*F*_2_ population (‘Sam’ × CN113536)					*F*_3_ population (‘Sam’ × CN113536)				
Powdery mildew isolate	Resistant	Susceptible	*χ*^2^_3:1_	*p* value (5%)	Resistant	Segregating	Susceptible	*χ*^2^_1:2:1_	*p* value (5%)
Białka	104	42	0.579	0.30	25	79	42	5.213	0.05
Laski	103	43	1.303	0.20	21	82	43	6.221	0.02

To identify silicoDArT markers for new resistance gene to oat powdery mildew, designed *Pm11*, F_2_ segregating population ‘Sam’ × *A. sterilis* CN113536 were analysed using DArTseq methodology. A total of 30,620 silicoDArT markers were identified: 29,538 markers were polymorphic, and 202 were highly correlated with resistance in the analysed population. Among them, based on the length of the sequence, 71 were selected for potential conversion: 42 specific to resistant and 29 specific to susceptible individuals. Finally, 40 silicoDArT markers were suitable for primer design. Two pairs of specific primers were proposed for each sequence. First, all designed specific primers were tested on a group of 5 resistant and 5 susceptible genotypes. From this pool, five markers were selected: 3 for resistant and 2 for susceptible plants, which initiated the amplification of the products in the expected groups (Table 2).

**Table 2:** Selected silicoDArT markers successfully converted into SCAR

SilicoDArT marker ID	SCAR primers	Type of genotype confirmed by a marker	Primers sequence	Primer TM	SCAR length	Correlation with phenotype observation (*p* < 0.05)
24031766	Pm11-3F Pm11-3R	Resistant	AACGTGCGGCCTCTA ACCATGCTCTAACGGAAA	57	55	0.592
5420825	Pm11-21F Pm11-21R	Resistant	AACCTGATAGTGACCAA CAGAGAAGTACGCCAA	51	51	0.513
3455968	Pm11-41F Pm11-41R	Resistant	GTGGAATTAATGTGCTGG ATCTCGGTCCTGCT	53	44	0.118
5425222	Pm11-48F Pm11-48R	Susceptible	CAGCCACACACACCTA GTGTCCCCTTGTATCTT	53	42	0.330
3280382	Pm11-49F Pm11-49R	Susceptible	GCGTCGTGCTGTATG CGTTGTTGTTCTTGTTTCTG	55	57	0.344

**Fig. 1:**
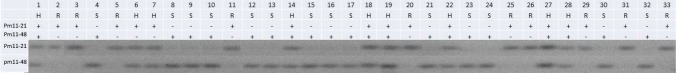
Molecular profiles obtained with Pm11-21 and Pm11-48 primer pairs for individuals and parental line of ‘Sam’ × CN113536 population. 1-31 individuals of the analysed population, 32—cultivar Sam, 33-*A. sterilis* CN113536

The selected primers were tested on the set of 92 genotypes that were subject to DArTseq genotyping to verify the correctness of the conversion process. The correlation of the converted markers and the original silicoDArT markers was very high, indicating that the marker conversion process was correct. In the next step, PCR with selected primers was carried out for all 146 individuals of the *F*_2_ population to calculate the correlation between the converted markers and the phenotypic observations, at a *p* value < 0.05 (Table 2). Markers specific to resistant plants initiated amplification of the products for both resistant homozygotes and heterozygotes. The correlation between the presence of the marker and the phenotype observation was 0.592 (Pm11-3F,R) and 0.513 (Pm11-21F,R); a very low correlation (0.118) was obtained for primer Pm11-41 F,R, and it was excluded from further analyses. Both markers were present in all resistant homozygotes: the marker Pm11-3 was present in 65 plants phenotyped as heterozygous, and Pm11-21 was present in 61 samples. The markers specific to susceptible genotypes (Pm11-48 and Pm11-49) initiated the amplification of products in susceptible homozygotes, and the expected products were also observed in individuals phenotyped in F_3_ as heterozygotes (Pm11-48 in 65 and Pm11-49 in 64). The results obtained from PCR and the host–pathogen tests allowed us to develop a good method of identifying resistant and susceptible genotypes in the analysed population. First, the application of the single markers Pm11-3 and Pm11-21 allowed the selection of resistant plants with new *Pm* gene. Second, the use of a combination of marker Pm11-21 (specific to resistant plants) and marker Pm11-48 (specific to susceptible plants) allowed the selection of homozygous and heterozygous genes from the analysed population. After PCR with the two pairs of selected primers, the 1–0 pattern (presence–absence of the PCR product) was observed for resistant homozygotes, while the 0–1 pattern was recorded for susceptible homozygotes. The presence of both amplification products (1–1 pattern) was observed for individuals that were phenotyped as heterozygotes (Fig. 1).

The obtained silicoDArT markers were used for chromosomal localization of the new resistance gene. BLASTn analysis revealed that only five out of 71 segregating silicoDArT markers had their counterparts on the consensus map. All five markers located within 2.9-cM-long region of Mrg12. SilicoDArT marker 13752573 (avgbs_cluster_4196.1.27) positioned on 14.1cM; 3279616 (avgbs_cluster_42260.1.12)—15.5cM; 22076538 (avgbs2_107266.1.50)—15.9cM; 5446415 (avgbs_218395)—17.0cM; and 5432233 (avgbs_104885)—17.0cM.

## Discussion

Thus far, 10 major genes conditioning resistance to oat powdery mildew have been characterized. Among the described *Pm* genes, *Pm1*, *Pm3* and *Pm6* are common in commercial cultivars from Europe and North America (Hsam et al. 1997, 1998; Okoń 2012; Okoń et al. 2016a). However, the resistance conditioned by these genes has already been broken by existing races of the pathogen, and only *Pm4* and *Pm7* are still effective (Okoń 2015). There is no available information about the effectiveness of two resistance genes recently identified by Herrmann and Volker (2018): *Pm9* and *Pm10* from *Avena byzantina*. The authors mentioned only that genotypes with these genes were resistant against a highly virulent powdery mildew isolate. These genes have not been used in breeding programmes thus far. Herrmann and Volker (2018) mentioned that *Pm7* is effective and successfully used in breeding programmes in Germany, but Okoń and Ociepa (2017) found isolates that were virulent to plants with the *Pm7* gene. The virulence of the pathogen population has changed, and new, more aggressive pathotypes have appeared. To maintain a high level of resistance, it is necessary to seek out and introduce new and effective resistance genes for breeding programmes.

Okoń and Ociepa (2018) found that *A. sterilis* genotype CN113536 possesses a new and highly effective gene against existing powdery mildew pathotypes. The presented work concerns the characterization of the inheritance of this new mildew resistance gene, its chromosomal location and the development of DNA markers, allowing its identification in the oat genome. The conducted host–pathogen tests showed that segregation of resistance in the *F*_2_ population was close to the 3:1 model and that in the *F*_3_ population segregation fits the 1:2:1 model. Based on these results, and results obtained by Okoń and Ociepa (2018), we postulate the presence of a new resistance gene in *A. sterilis* genotype CN113536. We named it *Pm11*.

According to the oat map published by Bekele et al. (2018), our new resistance gene (*Pm11*) was located on Mrg12. Only five out of 71 segregating silicoDArT markers had their counterparts on the consensus map. The reason is probably origin of these sequences from the *A. sterilis* CN113536—donor of powdery mildew resistance gene. The early generation of mapping population and a modest/minor number of recombination events could also contribute to that. Nevertheless, five markers positioned on the consensus map pointed at a very restricted region.

Chaffin et al. (2016) underline the fact that only nine merge chromosomes (Mrg) were assigned with high level of certainty to the corresponding chromosome assignments, inferred by Oliver et al. (2013). The authors discuss the fact that Mrg12 and chromosome 13A not be confirmed as the same. Moreover, they identified the strong homology between Mrg12 and Mrg02, which is assigned to chromosome 9D. Previous research conducted by Hsam et al. (2014) showed that the *Pm7* gene was located on the chromosome 13A. The host–pathogen tests presented in our previous work (Okoń and Ociepa 2018) showed significant differences in infection patterns between control genotypes with the *Pm7* and new *Pm11* gene. A similar situation was found by Herrmann and Volker (2018), who mapped the *Pm10* gene to chromosome 10D, on which the *Pm6* gene was present (Hsam et al. 2014). They also distinguished these two genes based on different infection patterns. Moreover, they suggested that no clear distinction between *Pm8* and *Pm10* can be presently obtained. Herrmann and Volker (2018) also found that the *Pm9* gene is located close to *Pm5*. These results suggest that *Pm*, similar to the *Pc* genes, exists in clusters in the genome, in groups that are either tightly linked or allelic (Kiehn et al. 1976; Harder and McKenzie 1980; Martens et al. 1980).

The introduction of new resistance genes into cultivated forms increases the level of cultivar resistance. However, the possibility of conducting selection based on molecular markers is a very important aspect of modern plant breeding. There are several examples of the use of molecular markers to identify important traits in oats, such as SNP markers associated with short straw (Tanhuanpää et al. 2006), AFLP and RAPD markers associated with flowering time in Brazilian oat varieties (Locatelli et al. 2006), SCAR and CAPS markers linked to ß-glucan and protein content (Orr and Molnar 2008) and AFLP markers linked to BYDV resistance (Jin et al. 1998). A certain percentage of molecular markers are associated with resistance to fungal disease, such as crown rust (Chen et al. 2006; Rines et al. 2018), stem rust (Penner et al. 1993) and powdery mildew (Okon et al. 2018a, b; Okoń and Kowalczyk 2012b; Yu and Herrmann 2006). Yu and Herrmann (2006) identified a co-dominant simple sequence repeat (SSR) marker and developed four AFLP-derived STS markers tightly linked to the *Pm5* resistance gene. Okoń and Kowalczyk (2012b) developed the SCAR-BG8 marker linked to *Pm6.* Molecular markers specific to *Pm4* were developed by Okon et al. (2018a, b) using DArTseq technology.

The current work attempted to develop a method for identifying a new powdery mildew resistance gene in oat based on the use of molecular markers obtained by PCR. Random markers associated with the *Pm11* gene have been identified by the DArTseq method. In our study, we used the silicoDArT method for more numerous markers. There is limited information in the available literature about the successful conversion of DArT markers. McCartney et al. (2011) mapped *Pc91* to a linkage group consisting of 44 DArTs. They developed five PCR-based markers that co-segregated with *Pc91* from three non-redundant DArTs. A study conducted by Okon et al. (2018a, b) also confirmed the possibility of converting DArTseq markers into PCR-specific markers, but only 3 primer pairs produced the expected patterns across the mapping population.

In the present study, the conversion of silicoDArT markers allows for the identification of genotypes with *Pm11* powdery mildew resistance gene. Also, the use of these markers allowed us to identify heterozygotes in the analysed population. The obtained markers can be used in the selection of genotypes with the *Pm11* gene as well as in the identification of homozygous and heterozygous genes in breeding programmes. These markers can also be useful in gene pyramid identification which is a very good way to achieve long-term resistance against powdery mildew in oat cultivars.

## References

[CR1] Aung T, Thomas H, Jones IT (1977). The transfer of the gene for mildew resistance from *Avena barbata* (4x) into the cultivated oat *A. sativa* by an induced translocation. Euphytica.

[CR01] Banyal DK, Sood VK, Singh A, Mawar R (2016). Integrated management of oat diseases in north-western Himalaya. Range Manag Agrofor.

[CR2] Barbosa MM, Federizzi LC, Milach SCK (2006). Molecular mapping and identification of QTL’s associated to oat crown rust partial resistance. Euphytica.

[CR3] Becher R (2007). EST-derived microsatellites as a rich source of molecular markers for oats. Plant Breed.

[CR4] Bekele WA, Wight CP, Chao S (2018). Haplotype-based genotyping-by-sequencing in oat genome research. Plant Biotechnol J.

[CR5] Bennett FG (1984). Resistance to powdery mildew in wheat: a review of its use in agriculture and breeding programmes. Plant Pathol.

[CR6] Bewick V, Cheek L, Ball J (2004). Statistics review 8: qualitative data—tests of association. Crit Care.

[CR7] Chaffin AS, Huang Y-F, Smith S (2016). A consensus map in cultivated hexaploid oat reveals conserved grass synteny with substantial subgenome rearrangement. Plant Genome.

[CR8] Chen G, Chong J, Gray M (2006). Identification of single-nucleotide polymorphisms linked to resistance gene *Pc68* to crown rust in cultivated oat. Can J Plant Pathol.

[CR9] Collard BCY, Mackill DJ (2008). Marker-assisted selection: an approach for precision plant breeding in the twenty-first century. Philos Trans R Soc Lond B Biol Sci.

[CR10] Dean R, Van Kan JAL, Pretorius ZA (2012). The top 10 fungal pathogens in molecular plant pathology. Mol Plant Pathol.

[CR11] Harder DE, McKenzie RIH (1980). Inheritance of crown rust resistance in three accessions of *Avena sterilis*. Can J Genet Cytol.

[CR12] Hau B, de Vallavieille-Pope C, Cooke BM, Gareth Jones D, Kaye B (2006). Wind-dispersed diseases. The epidemiology of plant diseases.

[CR13] Herrmann MH, Mohler V (2018). Locating two novel genes for resistance to powdery mildew from *Avena byzantina* in the oat genome. Plant Breed.

[CR14] Hoppe HD, Kummer M (1991) New productive hexaploid derivatives after introgression of *Avena pilosa* features. In: Cereal breeding: Eucarpia cereal section meeting, Schwerin (Germany), 24–27 Jun 1991, pp 56–61

[CR15] Hsam SLK, Peters N, Paderina EV (1997). Genetic studies of powdery mildew resistance in common oat (*Avena sativa* L.) I. Cultivars and breeding lines grown in Western Europe and North America. Euphytica.

[CR16] Hsam SLK, Paderina EV, Gordei S, Zeller FJ (1998). Genetic studies of powdery mildew resistance in cultivated oat (*Avena sativa* L.) II. Cultivars and breeding lines grown in Northern and Eastern Europe. Hereditas.

[CR17] Hsam SLK, Mohler V, Zeller FJ (2014). The genetics of resistance to powdery mildew in cultivated oats (*Avena sativa* L.): current status of major genes. J Appl Genet.

[CR18] Jin H, Domier LL, Kolb FL, Brown CM (1998). Identification of quantitative loci for tolerance to barley yellow dwarf virus in oat. Phytopathology.

[CR19] Jones IT, Roderick HW, Clifford BC (1987). The integration of host resistance with fungicides in the control of oat powdery mildew. Ann Appl Biol.

[CR20] Kiehn FA, McKenzie RIH, Harder DE (1976). Inheritance of resistance to *Puccinia coronata avenae* and its association with seed characteristics in four accessions of *Avena sterilis*. Can J Genet Cytol.

[CR21] Kilian B, Graner A (2012). NGS technologies for analyzing germplasm diversity in genebanks. Brief Funct Genom.

[CR22] Lawes DA, Hayes JD (1965). The effect of mildew (*Erysiphe graminis* f.SP. *avenae*) on spring oats. Plant Pathol.

[CR23] Leath S (1991). Reaction of winter oat germ plasm to an epidemic of oat powdery mildew. Plant Dis.

[CR24] Li CD, Rossnagel BG, Scoles GJ (2000). The development of oat microsatellite markers and their use in identifying relationships among *Avena* species and oat cultivars. TAG Theor Appl Genet.

[CR25] Locatelli AB, Federizzi LC, Milach SCK (2006). Loci affecting flowering time in oat under short-day conditions. Genome.

[CR26] Martens JW, McKenzie RIH, Harder DE (1980). Resistance to *Puccinia graminis avenae* and *P. coronate avenae* in the wild and cultivated *Avena* populations of Iran, Iraq, and Turkey. Can J Genet Cytol.

[CR27] Martinelli J (2004) Oat diseases and their control. In: Suttie J, Reynolds S (eds) Fodder oats: a world overview. Food and Agriculture Organization of The United Nations, Rome, pp 197–214

[CR28] McCartney CA, Stonehouse RG, Rossnagel BG (2011). Mapping of the oat crown rust resistance gene *Pc91*. Theor Appl Genet.

[CR29] Milczarski P, Hanek M, Tyrka M, Stojałowski S (2016). The application of GBS markers for extending the dense genetic map of rye (*Secale cereale* L.) and the localization of the Rfc1 gene restoring male fertility in plants with the C source of sterility-inducing cytoplasm. J Appl Genet.

[CR30] Morikawa T (1995). Transfer of mildew resistance from the wild oat *Avena prostrata* into the cultivated oat. Bull Univ Osaka Prefect Ser B.

[CR31] Okoń SM (2012). Identification of powdery mildew resistance genes in Polish common oat (*Avena sativa* L.) cultivars using host-pathogen tests. Acta Agrobot.

[CR32] Okoń SM (2015). Effectiveness of resistance genes to powdery mildew in oat. Crop Prot.

[CR33] Okoń S, Kowalczyk K (2012). Deriving isolates of powdery mildew (*Blumeria graminis* DC. f.sp. *avenae* Em. Marchal.) in common oat (*Avena sativa* L.) and using them to identify selected resistance genes. Acta Agrobot.

[CR34] Okoń S, Kowalczyk K (2012). Identification of SCAR markers linked to resistance to powdery mildew in common oat (*Avena sativa*). J Plant Dis Prot.

[CR35] Okoń SM, Ociepa T (2017). Virulence structure of the *Blumeria graminis* DC. f. sp. *avenae* populations occurring in Poland across 2010–2013. Eur J Plant Pathol.

[CR36] Okoń SM, Ociepa T (2018). Effectiveness of new sources of resistance against oat powdery mildew identified in *A. sterilis*. J Plant Dis Prot.

[CR37] Okoń S, Ociepa T, Paczos-Grzęda E, Kowalczyk K (2016). Analiza poziomu odporności polskich odmian owsa zwyczajnego (*Avena sativa* L.) na mączniaka prawdziwego (*Blumeria graminis* DC. f. sp. *avenae* Em. Marchal.). Ann UMCS Sect E Agric.

[CR38] Okoń S, Paczos-Grzęda E, Ociepa T (2016). *Avena sterilis* L. genotypes as a potential source of resistance to oat powdery mildew. Plant Dis.

[CR39] Okoń S, Ociepa T, Nucia A (2018). Molecular Identification of Pm4 powdery mildew resistant gene in oat. Not Bot Hortic Agrobot Cluj-Napoca.

[CR40] Okoń S, Ociepa T, Paczos-Grzęda E, Ladizinsky G (2018). Evaluation of resistance to *Blumeria graminis* (DC.) f. sp. *avenae*, in *Avena murphyi* and *A. magna* genotypes. Crop Prot.

[CR41] Oliver RE, Tinker NA, Lazo GR (2013). SNP discovery and chromosome anchoring provide the first physically-anchored hexaploid oat map and reveal synteny with model species. PLoS ONE.

[CR42] Orr W, Molnar SJ (2008). Development of PCR-based SCAR and CAPS markers linked to β-glucan and protein content QTL regions in oat. Genome.

[CR43] Paczos-Grzęda EM, Bednarek PT, Koroluk A (2014). Avena sativa L. intercultivar polymorphism assessment using silicoDArT markers./Zastosowanie markerów silicoDArT do oceny polimorfizmu międzyodmianowego. Folia Pomeranae Univ Technol Stetin Agric Aliment Piscaria Zootech.

[CR44] Pal N, Sandhu JS, Domier LL, Kolb FL (2002). Development and characterization of microsatellite and RFLP-derived PCR markers in oat. Crop Sci.

[CR45] Penner GA, Chong J, Lévesque-Lemay M (1993). Identification of a RAPD marker linked to the oat stem rust gene *Pg3*. Theor Appl Genet.

[CR46] Rines HW, Miller ME, Carson M (2018). Identification, introgression, and molecular marker genetic analysis and selection of a highly effective novel oat crown rust resistance from diploid oat, *Avena strigosa*. Theor Appl Genet.

[CR47] Roderick HW, Jones ERL, Sebesta J (2000). Resistance to oat powdery mildew in Britain and Europe: a review. Ann Appl Biol.

[CR48] Sánchez-Martín J, Rubiales D, Prats E (2011). Resistance to powdery mildew (*Blumeria graminis* f. sp. *avenae*) in oat seedlings and adult plants. Plant Pathol.

[CR49] Schwarzbach E, Smith IM, Smith IM, Dunez J, Lelliot RA (1988). *Erysiphe graminis* DC. European handbook of plant diseases.

[CR50] StatSoft (2017) STATISTICA (data analysis software system), version 13.1

[CR51] Stevens EJ, Armstrong KW, Bezar HJ, Suttie JM, Reynolds SG (2004). Fodder oats: an overview. Fodder oats: an overview.

[CR52] Takamatsu S (2004). Phylogeny and evolution of the powdery mildew fungi (Erysiphales, Ascomycota) inferred from nuclear ribosomal DNA sequences. Mycoscience.

[CR53] Tanhuanpää P, Kalendar R, Laurila J (2006). Generation of SNP markers for short straw in oat (*Avena sativa* L.). Genome.

[CR54] Thomas H, Marshall HG, Sorrells ME (1992). Cytogenetics of *avena*. Oat science and technology.

[CR55] Xue LH, Li CJ, Zhao GQ (2017). First report of powdery mildew caused by *Blumeria graminis* on *Avena sativa* in China. Plant Dis.

[CR56] Yu J, Herrmann M (2006). Inheritance and mapping of a powdery mildew resistance gene introgressed from *Avena macrostachya* in cultivated oat. Theor Appl Genet.

